# Effects of Different Culture Conditions on the Synthesis and Distribution of Polyunsaturated Fatty Acids (EPA and ARA) in *Porphyridium purpureum*

**DOI:** 10.3390/md24030114

**Published:** 2026-03-19

**Authors:** Tao Li, Bingqi Xu, Yiyang Wu, Liang Wei, Hualian Wu, Houbo Wu, Wenzhou Xiang, Jin Xu

**Affiliations:** 1 State Key Laboratory of Breeding Biotechnology and Sustainable Aquaculture, Guangdong Key Laboratory of Marine Materia Medica, South China Sea Institute of Oceanology, Chinese Academy of Sciences, Guangzhou 510301, China; taoli@scsio.ac.cn (T.L.); xubingqi24@mails.ucas.ac.cn (B.X.); wuyiyang25@mails.ucas.ac.cn (Y.W.); weiliang23@mails.ucas.ac.cn (L.W.); hlwu@scsio.ac.cn (H.W.); wuhoubo@scsio.ac.cn (H.W.); 2 Guangdong Provincial Key Laboratory of New and Renewable Energy Research and Development, CAS Key Laboratory of Renewable Energy, Guangzhou Institute of Energy Conversion, Chinese Academy of Sciences, Guangzhou 510640, China

**Keywords:** *Porphyridium*, culture conditions, lipids, EPA and ARA, fatty acid distribution

## Abstract

The arachidonic acid (C20:4 ω6, ARA) and eicosapentaenoic acid (C20:5 ω3, EPA) from *Porphyridium purpureum* endow this microalga with potential utilization value, but their distribution patterns remain poorly understood. In this study, a nitrogen concentration, a phosphorus concentration, light intensity and salinity were applied to investigate the synthesis and distribution patterns of EPA and ARA in *P. purpureum* by measuring growth, lipid content, lipid fractions, fatty acid composition, and the levels of EPA and ARA in storage lipids and membrane lipids. The results show that the optimal conditions for biomass accumulation were a nitrogen concentration of 0.75 g L^−1^, a phosphorus concentration of 240 mg L^−1^, a light intensity of 250–300 μmol photons m^−2^ s^−1^ and a salinity of 50 ppt. Reducing the phosphorus concentration and increasing salinity enhanced the total lipid content, whereas changes in nitrogen concentration and light intensity had minimal effects on total lipid content. Low nitrogen concentration, low phosphorus concentration and high light intensity favored ARA synthesis, whereas the opposite conditions promoted EPA synthesis. Culture conditions could alter the distribution of ARA and EPA between storage lipids and membrane lipids. Increasing the nitrogen concentration, phosphorus concentration and salinity, as well as reducing light intensity, promoted the distribution of ARA and EPA in membrane lipids. Conversely, the opposite conditions enhanced their distribution in storage lipids. In conclusion, the synthesis and distribution of EPA and ARA in *P. purpureum* are influenced by culture conditions. To improve the yield of ARA and EPA, *P. purpureum* should be cultivated under nutrient-sufficient conditions.

## 1. Introduction

*Porphyridium purpureum***,** a unicellular red microalga, is capable of synthesizing high-value bioactive compounds such as B-phycoerythrin, long-chain polyunsaturated fatty acids (PUFAs) and sulfated exopolysaccharides [1]. The arachidonic acid (C20:4, ARA) and eicosapentaenoic acid (C20:5, EPA) produced by *P. purpureum* have garnered significant interest owing to their important physiological functions, such as cardiovascular disease prevention, anti-inflammatory effects, promotion of brain development and immune regulation [1]. Beyond their medical significance, ARA and EPA serve critical roles in animal nutrition (aquaculture and feed) and agriculture as biostimulants to improve crop yield and stress tolerance [2,3]. In microalgal cells, ARA and EPA are primarily esterified into triacylglycerols (TAGs), phospholipids and glycolipids, rather than existing as free fatty acids. Within these lipids, they fulfill critical biological roles, such as storing energy and maintaining membrane structure [4,5]. The form of EPA and ARA significantly influences their absorption and utilization in humans. For instance, phospholipid-bound EPA, particularly phosphatidylcholine-bound EPA, was integrated more rapidly into erythrocyte membranes compared to triglyceride-bound EPA, making it more readily absorbed. This advantage of phospholipid-bound EPA arises primarily from the emulsifying and solubilizing properties of phospholipids, coupled with the preferential hydrolysis of fatty acids located at the sn-2 position [6]. Therefore, elucidating the forms and synthesis patterns of ARA and EPA in *P. purpureum* is crucial for advancing their downstream applications.

The lipids synthesized by *Porphyridium* can be categorized into storage lipids (TAGs) and membrane lipids (glycolipids, phospholipids and sphingolipids) [4]. These perform distinct intracellular physiological functions—storage lipids serve as reserves of energy and carbon sources, while membrane lipids are primarily structural components of cell membranes and thylakoid membranes. Some oleaginous microalgae can synthesize large amounts of storage lipids under stress conditions [7]. In contrast, membrane lipids exhibit stability in their proportions owing to their critical physiological functions. Given the limited capacity for lipid accumulation in *Porphyridium* [8], the changes in the composition of lipids and fatty acids remain poorly reported in the literature.

To understand the distribution patterns of EPA and ARA in different glycerolipids, an overview of their biosynthetic pathways is required. Acetyl-CoA serves as the precursor for the de novo synthesis of fatty acids, leading to the formation of C16:0, C18:0 and C18:1. Subsequently, these are converted into EPA and ARA through a series of reactions catalyzed by fatty acid desaturases and elongases. Fatty acid desaturases preferentially utilize phospholipids (e.g., phosphatidylcholine) as substrates rather than free fatty acids [9]. On the other hand, fatty acid chain elongases primarily use acyl-CoA as substrates. In microalgae, the distribution of fatty acids among different lipid classes is mainly determined by differences in the subcellular localization and substrate specificity of fatty acid desaturases [10]. Culture conditions can influence the synthesis of EPA and ARA, thereby affecting their content in biomass. For example, nitrogen starvation could increase EPA content in *Nannochloropsis oculata* [11], while nitrogen limitation enhanced ARA content in *Porphyridium cruentum* CCALA 415 [4]. In *Phaeodactylum tricornutum*, the percentage of EPA decreased significantly as temperature increased from 15 °C to 25 °C. In addition to genetic regulation, the distribution of EPA and ARA among different lipid classes is also influenced by culture conditions. For instance, temperature variations have been reported to change the content of ARA and EPA in lipid classes such as monogalactosyldiaclyglycerol (MGDG), digalactosyldiaclyglycerol (DGDG), sulfoquinovosyldiacylglycerol (SQDG), phosphatidylcholine (PC), phosphatidylglycerol (PG) and TAG in *Nannochloropsis* [12]. However, such changes have been less documented in *Porphyridium*. To date, only one study, published in 2024, investigated the synthesis pathways of PUFAs in *Porphyridium* under two nitrogen concentrations by using lipidomics and transcriptomics. That study demonstrated that nitrogen limitation can alter the distribution of EPA and ARA among different lipid molecular species [4].

The commonly controllable cultivation conditions of microalgae include the nitrogen concentration, phosphorus concentration, light intensity and salinity [13], which are also frequently used as means to induce the accumulation of the secondary metabolite. Under nitrogen limitation and high-light conditions, *Haematococcus pluvialis* accumulates astaxanthin [14], *Dunaliella salina* synthesizes beta-carotene [15] and oleaginous microalgae produce storage lipids [16]. Nitrogen limitation significantly increased the total lipid content (predominantly in the form of storage lipids, i.e., TAGs) in *Porphyridium*; it also enhanced ARA synthesis but inhibited EPA synthesis [4]. It also altered the distribution of ARA and EPA among different lipid molecular species. However, there were too few nitrogen concentration groups in the experiment [4]. The thylakoid membrane is the main location where the initial photosynthetic reactions occur. Glycolipids are major components of the thylakoid membrane. In order to adapt to different light intensities, microalgae cells typically adjust the fatty acid composition of membrane lipids. For example, *Pavlova lutheri* enhanced the synthesis of thylakoid membranes (mainly glycolipids) to capture more light under low-light conditions [17]. The salinity of the culture medium can affect the osmotic pressure inside and outside the cell. Under high-salinity conditions, microalgae adapt to osmotic changes by increasing the proportion of PUFAs of the membranes, thereby enhancing membrane fluidity [18]. The above results suggested that under different culture conditions, the lipid composition, fatty acid content and their distribution in *Porphyridium* are likely to undergo changes.

The aim of this study was to elucidate the synthesis and distribution patterns of ARA and EPA in *Porphyridium* in response to key cultivation factors, to guide its targeted cultivation for PUFA production. To achieve this, the following objectives were pursued: (1) to analyze the lipid profiles (including total lipids, storage/membrane lipid distribution and fatty acid composition) in response to varying nitrogen, phosphorus, light and salinity conditions; (2) to specifically track the content and distribution of ARA and EPA between storage and membrane lipid pools under these conditions. The study aims to provide a strategy for the targeted production of desired lipid products.

## 2. Results

### 2.1. Effects of Culture Conditions on the Growth of P. purpureum SCS-02

**Figure 1:**
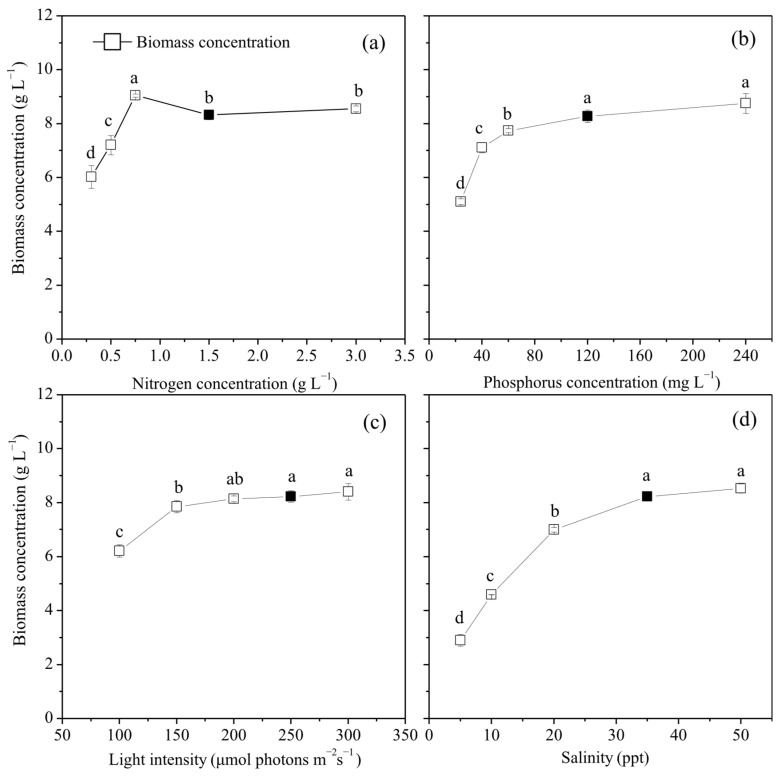
Biomass concentration of *Porphyridium purpureum* SCS-02 under different culture conditions: (**a**) Nitrogen concentration; (**b**) phosphorus concentration; (**c**) light intensity; (**d**) salinity. The black squares represent the control group. The values shown are the averages of three biological replicates and three technical replicates ± standard deviation. Different letters denote significant differences in biomass concentration under different culture conditions (level of significance, *p* < 0.05).

As shown in Figure 1, the highest biomass of SCS-02 was obtained under the nitrogen concentration of 0.75 g L^−1^, reaching 7.87 g L^−1^ among all nitrogen concentration groups. Biomass also increased with rising phosphorus concentration, light intensity, and salinity, attaining the maximum at 300 μmol photons m^−2^ s^−1^, 240 mg L^−1^ and 50 ppt, respectively. The maximum biomass was 9.77 g L^−1^, 9.73 g L^−1^ and 8.83 g L^−1^, respectively. Thus, the optimal conditions for biomass accumulation in SCS-02, when considering each factor independently, are a nitrogen concentration of 0.75 g L^−1^, a phosphorus concentration of 240 mg L^−1^, a light intensity of 300 μmol photons m^−2^ s^−1^ and a salinity of 50 ppt.

### 2.2. Effects of Culture Conditions on the Accumulation of Total Lipids of P. purpureum SCS-02

**Figure 2:**
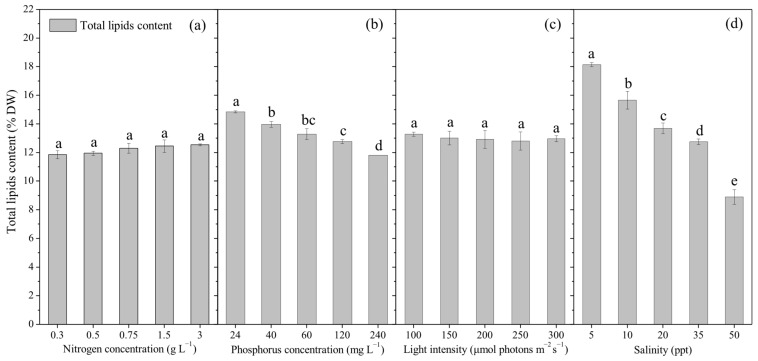
Lipid accumulation of *Porphyridium purpureum* SCS-02 under different culture conditions: (**a**) Nitrogen concentration; (**b**) phosphorus concentration; (**c**) light intensity; (**d**) salinity. The values shown are the averages of three biological replicates and three technical replicates ± standard deviation. Different letters denote significant differences among different treatment groups (level of significance, *p* < 0.05).

Figure 2a indicates that nitrogen concentrations had minimal effect on the total lipid content of SCS-02, with values ranging from 11.9% to 12.6% dry weight (DW). Although a slight increasing trend was observed, the increase was only 0.7% DW (*p* > 0.05). In contrast, increasing phosphorus concentration led to a gradual decline in total lipid content (Figure 2b). The highest total lipid content (14.8% DW) was achieved at 24 mg L^−1^ phosphorus concentration, while the lowest (11.8% DW) occurred at 240 mg L^−1^ phosphorus concentration, representing a significant decrease of 3.0% DW (*p* < 0.05). Figure 2c shows that light intensity had little influence on total lipid content, with all treatment groups ranging from 12.8% to 13.3% DW—a difference of just 0.5% DW (*p* > 0.05). However, Figure 2d reveals that salinity had a significant effect: high salinity resulted in high total lipid content. At a salinity of 50 ppt, the total lipid content reached 18.1% DW, compared to only 9.9% DW at 5 ppt, a significant variation of 8.2% DW (*p* < 0.05). These findings suggest that low phosphorus and high salinity increased total lipid content in SCS-02, whereas nitrogen concentration and light intensity had negligible effects.

### 2.3. Effects of Cultivation Conditions on the Lipid Fractionation of P. purpureum SCS-02

**Figure 3:**
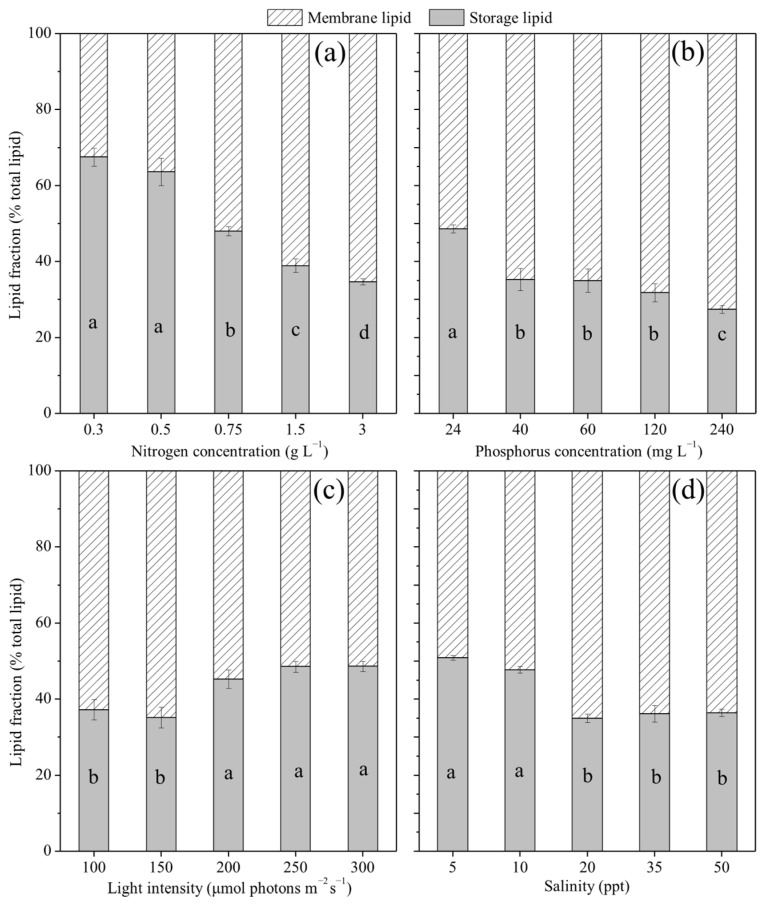
Lipid fractionation of *Porphyridium purpureum* SCS-02 under different culture conditions: (**a**) Nitrogen concentration; (**b**) phosphorus concentration; (**c**) light intensity; (**d**) salinity. The values shown are the averages of three biological replicates and three technical replicates ± standard deviation. Different letters denote significant differences among different treatment groups (level of significance, *p* < 0.05).

Figure 3a demonstrates that nitrogen concentration had a significant effect on the lipid fractionation of SCS-02. As nitrogen concentration increased from 0.3 to 3.0 g L^−1^, the proportion of membrane lipids rose from 32.5% to 65.3% of total lipids (TL), representing a 101.1% increase (*p* < 0.05), while the proportion of storage lipids declined from 67.5% to 34.7% TL, representing a 48.7% decrease (*p* < 0.05). Figure 3b shows that increasing phosphorus concentration from 24 to 240 mg L^−1^ led to a rise in the proportion of membrane lipids from 51.4% to 72.6% TL (41.3% increase, *p* < 0.05), and a decrease in the proportion of storage lipids from 48.6% to 27.4% TL (43.6% decrease, *p* < 0.05). In Figure 3c, increasing light intensity from 100 to 300 μmol photons m^−2^ s^−1^ resulted in a reduction in the proportion of membrane lipids from 62.8% to 51.3% TL (18.2% decrease, *p* < 0.05), while the proportion of storage lipids increased from 37.3% to 48.7% TL (30.7% increase, *p* < 0.05). Figure 3d indicates that as salinity increased from 5 to 50 ppt, the proportion of membrane lipids increased from 49.1% to 63.6% TL (29.6% increase, *p* < 0.05), whereas the proportion of storage lipids decreased from 50.9% to 36.4% TL (28.5% decrease, *p* < 0.05). Overall, these results indicate that elevated nitrogen and phosphorus concentrations, as well as increased salinity, promoted membrane lipid synthesis but inhibited storage lipid synthesis. In contrast, increased light intensity favors storage lipid accumulation while reducing membrane lipid synthesis.

### 2.4. Effects of Cultivation Conditions on the Fatty Acid Composition of P. purpureum SCS-02

**Figure 4:**
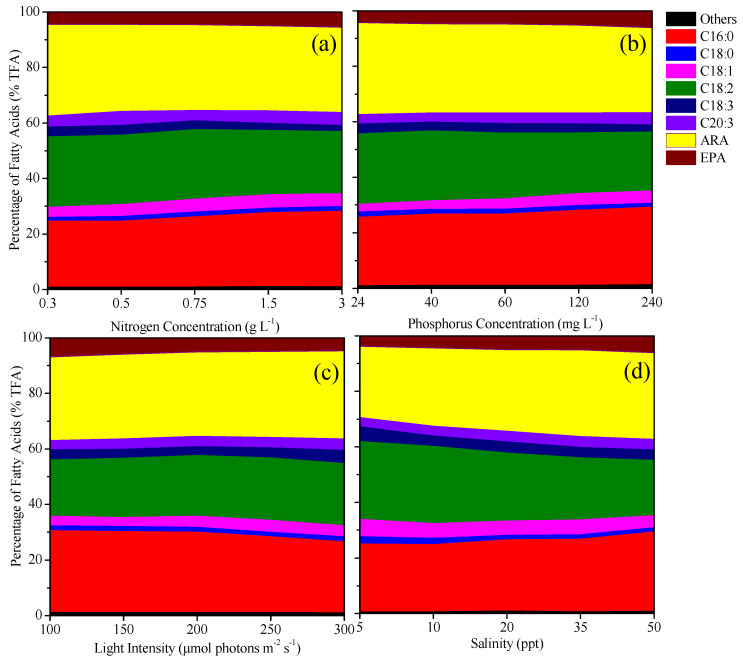
Change in fatty acid composition of *Porphyridium purpureum* SCS-02 under different culture conditions: (**a**) Nitrogen concentration; (**b**) phosphorus concentration; (**c**) light intensity; (**d**) salinity. TFA: Total fatty acids; EPA: eicosapentaenoic acid; ARA: arachidonic acid. The values shown are the averages of three biological replicates and three technical replicates ± standard deviation.

As shown in Figure 4, the fatty acid profile of SCS-02 included C16:0, C18:0, C18:1, C18:2, C18:3, C20:3 ω6, ARA and EPA. The most abundant fatty acids were ARA (25.8–32.8% total fatty acids, TFA), C16:0 (23.8–29.4% TFA) and C18:2 (18.3–28.0% TFA). Other fatty acids, including EPA (3.7–6.9% TFA), composed less than 10% of TFA. Increasing the nitrogen (0.3 to 3.0 g L^−1^) and phosphorus (24 to 240 mg L^−1^) concentrations led to a decrease in the percentage of ARA by 8.1% (*p* < 0.05) and 7.3% (*p* > 0.05), respectively. Conversely, increasing light intensity (100 to 300 μmol photons m^−2^ s^−1^) and salinity (5 to 50 ‰) resulted in increases in the percentage of ARA by 5.4% (*p* < 0.05) and 21.2% (*p* < 0.05), respectively. The percentage of EPA increased by 23.1%, 41.3% and 64.5% with rising nitrogen and phosphorus concentrations and salinity, respectively (*p* < 0.05), but decreased by 32.4% as light intensity increased (*p* < 0.05). C16:0 exhibited a similar trend to EPA, whereas C18:2 showed an opposite pattern. Specifically, the percentage of C18:2 decreased by 12.9%, 16.0% and 28.7% as nitrogen and phosphorus concentrations and salinity increased, respectively (*p* < 0.05), but increased by 10.4% with rising light intensity (*p* < 0.05). These findings indicated that reducing nitrogen and phosphorus concentrations and increasing light intensity and salinity enhance ARA synthesis. In contrast, increasing nitrogen and phosphorus concentrations and decreasing light intensity and salinity favored EPA synthesis.

### 2.5. Effects of Culture Conditions on the Distribution of EPA and ARA of P. purpureum SCS-02

**Figure 5:**
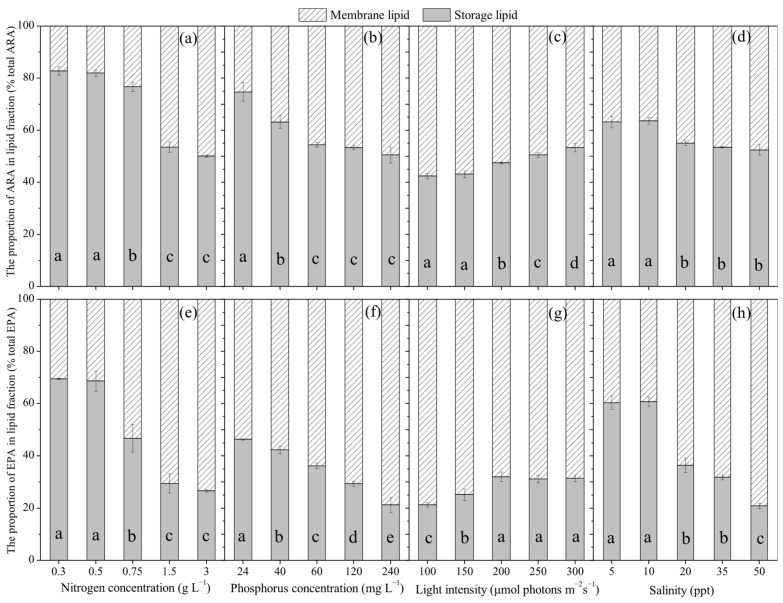
Proportion of ARA and EPA in different lipid fractions of *Porphyridium purpureum* SCS-02 under different culture conditions: (**a**,**e**) Nitrogen concentration; (**b**,**f**) phosphorus concentration; (**c**,**g**) light intensity; (**d**,**h**) salinity. EPA: eicosapentaenoic acid; ARA: arachidonic acid. The values shown are the averages of three biological replicates and three technical replicates ± standard deviation. Different letters denote significant differences among different treatment groups (level of significance, *p* < 0.05).

Under different nitrogen concentrations, the proportion of ARA in storage lipids ranged from 50.1% to 87.8% of total ARA, while in membrane lipids it ranged from 17.2% to 50.0% (Figure 5a). For EPA, the proportion in storage lipids ranged from 26.7% to 69.4%, and in membrane lipids from 30.6% to 73.4% of total EPA (Figure 5e). As nitrogen concentration increased, the proportion of ARA and EPA in storage lipids decreased by 39.5% (*p* < 0.05) and 61.6% (*p* < 0.05), respectively. Conversely, the proportion of ARA and EPA in membrane lipids increased by 189.8% (*p* < 0.05) and 140.1% (*p* < 0.05), respectively.

Figure 5b,f present the distribution of ARA and EPA between storage and membrane lipids under varying phosphorus concentrations. The proportion of ARA in storage lipids ranged from 50.5% to 74.8%, while in membrane lipids it ranged from 25.3% to 49.5% of the total ARA (Figure 5b). For EPA, the proportion in storage lipids ranged from 21.2% to 46.3%, and in membrane lipids from 53.7% to 78.8% of total EPA (Figure 5f). As phosphorus concentration increased, the proportion of ARA and EPA in storage lipids decreased by 32.4% (*p* < 0.05) and 54.2% (*p* < 0.05), respectively. In contrast, the proportion of ARA and EPA in membrane lipids increased by 95.9% (*p* < 0.05) and 46.8% (*p* < 0.05), respectively.

As shown in Figure 5c,g, the distribution of ARA and EPA between storage and membrane lipids was affected by light intensity. The proportion of ARA in storage lipids ranged from 42.4% to 53.3%, while in membrane lipids it ranged from 46.7% to 57.6% of the total ARA (Figure 5c). For EPA, the proportion in storage lipids ranged from 21.3% to 31.5%, and in membrane lipids from 68.5% to 78.7% of total EPA (Figure 5g). As light intensity increased, the proportion of ARA and EPA in storage lipids increased by 25.8% (*p* < 0.05) and 47.8% (*p* < 0.05), respectively. In contrast, the proportion of ARA and EPA in membrane lipids decreased by 19.0% (*p* < 0.05) and 12.9% (*p* < 0.05), respectively.

Figure 5d,h displays the distribution of ARA and EPA between storage and membrane lipids under different salinity conditions. The proportion of ARA in storage lipids ranged from 52.5% to 63.2%, while in membrane lipids it ranged from 36.8% to 47.5% of the total ARA (Figure 5d). For EPA, the proportion in storage lipids ranged from 20.9% to 60.3%, and in membrane lipids from 39.7% to 79.1% of total EPA (Figure 5h). As salinity increased, the proportion of ARA in storage lipids decreased by 17.0% (*p* < 0.05), while the proportion in membrane lipids increased by 29.1% (*p* < 0.05). Likewise, the proportion of EPA in storage lipids decreased by 65.4% (*p* < 0.05), while the proportion in membrane lipids increased by 99.4% (*p* < 0.05).

The above results indicated that ARA was more abundant than EPA in storage lipids, whereas EPA was more abundant than ARA in membrane lipids. Cultivation conditions substantially influenced the distribution of ARA and EPA between these lipid fractions. Specifically, increasing nitrogen and phosphorus concentrations, reducing light intensity, and increasing salinity led to high proportions of ARA and EPA in membrane lipids. Conversely, decreasing nitrogen and phosphorus concentrations, increasing light intensity, and lowering salinity increased the proportions of ARA and EPA in storage lipids.

### 2.6. Effect of Culture Conditions on EPA and ARA Content of P. purpureum SCS-02

As shown in Table 1, the contents of ARA and EPA displayed opposite trends. Low nitrogen, low phosphorus and high light intensity resulted in high ARA content and low EPA content, while high nitrogen, high phosphorus and low-light conditions led to high EPA content and low ARA content. The highest ARA content (4.43% DW) was observed in the 24 mg L^−1^ low phosphorus group and the highest EPA content (0.82% DW) was found in the 100 μmol photons m^−2^ s^−1^ light group. However, when assessing the production potential of EPA and ARA, the yield is also a critical indicator. High content does not always equate to high yield. For example, although the highest ARA content was observed at 24 mg L^−1^ phosphorus concentration, the highest yield occurred at 120 mg L^−1^ phosphorus concentration. This was because phosphorus limitation stimulated ARA synthesis but restricted microalgal growth, ultimately reducing ARA yield. The highest ARA yield and EPA yield were 18.84 mg L^−1^ and 3.49 mg L^−1^ among all the treatments. It was concluded that although ample nutrients and moderate salinity were essential for the biosynthesis of both ARA and EPA, their optimal light requirements were divergent. Specifically, a high light intensity promoted ARA production, in contrast to EPA, which was enhanced under low light intensity.

## 3. Discussion

Microalga oil has garnered great attention due to its various bioactive properties, including antioxidant, antibacterial, blood-lipid-lowering, neurodevelopment-promoting and immune-enhancing effects. The variations in its lipid fractionation and fatty acids contribute to the varying biologic activities of microalga oil [19]. Microalga oil rich in TAGs with high percentages of C16 and C18 fatty acids, such as those derived from *Chlorella* and *Nannochloropsis*, are suitable for biodiesel production [16]; whereas microalga oil, abundant in PUFAs like EPA and DHA, is considered the optimal feedstock for healthcare products and dietary supplements [20]. Recent studies indicated that the human body exhibited significant differences in the absorption of various lipid molecules. For instance, EPA oil extracted from *Nannochloropsis* demonstrated superior efficacy in lowering blood lipids compared to DHA microalga oil derived from *Schizochytrium* sp. [21]. Additionally, lipids extracted from *Chlorella* exhibited suppressive activities against lipase and inflammatory mediators, suggesting potential applications in acne treatment [22]. The fatty acid compositions and lipid fractionations of microalga oils can be changed by culture conditions [4,5]. Therefore, compared to the drawbacks of genetic modification, such as its lengthy cycle and the instability of microalga strains, achieving targeted production of microalgal lipids by modifying culture strategies may be an ideal approach.

The present study indicates that the total lipid content of *P. purpureum* SCS-02 ranged between 9.9% and 18.1% DW, which was notably lower than that of oleaginous microalgae such as *Nannochloropsis* and *Eustigmatophyceae* (over 40% DW), which was consistent with numerous previous studies on *Porphyridium* [4,23,24]. A lot of studies showed that reducing nitrogen concentration, lowering phosphorus levels and increasing light intensity could promote lipid accumulation in oleaginous microalgae [16]. The result of this study showed that only reducing phosphorus concentration slightly increased the total lipid content in *P. purpureum*, while lowering nitrogen concentration and increasing light intensity had minimal effects on its total lipid accumulation. This discrepancy might be attributed to the fact that carbohydrates, rather than storage lipids, serve as the carbon and energy storage in *P. purpureum*. Nitrogen reduction significantly enhanced carbohydrate accumulation in *P. purpureum* [8]. The elevated salinity increased the total lipid content of SCS-02 by 82.8%, which might be similar to the mechanism observed in other microalgae under high salinity levels. High salinity induced the intracellular generation of reactive oxygen species (ROS) such as superoxide (O_2_^−^) and hydrogen peroxide (H_2_O_2_), leading to membrane lipid peroxidation damage. In response, *P. purpureum* SCS-02 synthesized new phospholipids and glycolipids to repair the membrane, thus increasing the total lipid content [25].

Total lipid content is a rough indicator representing the sum of all liposoluble substances within cells. In this study, column chromatography was employed to separate storage lipids and membrane lipids based on differences in their polarity. The results show that the conditions conducive to membrane lipid synthesis included high nitrogen concentration, high phosphorus concentration, low light intensity and high salinity, whereas the conditions conducive to storage lipid synthesis included low nitrogen concentration, low phosphorus concentration, high light intensity and low salinity. These findings aligned with the lipid accumulation characteristics observed in many oleaginous microalgae; nitrogen starvation, phosphorus starvation and low NaCl concentrations enhanced neutral lipid contents [26]. Lowering the light intensity decreased the triglyceride content from 11.1 to 1.5% DW and increased the galactolipid content from 3.1 to 5.1% DW [27]. Increasing the NaCl concentration from 2.0 to 8.0 g L^−1^ resulted in a rising trend in total lipid content in *Chlorella*, with an increase of 53.1% compared to the control groups [28]. Under nitrogen and phosphorus limitation conditions, the synthesis of storage lipids can be activated in most microalgae [16]. Storage lipids are not the primary energy storage of *P. purpureum*. Although the proportion of storage lipids increased, the content of storage lipids did not rise significantly. This was also reflected in the results that nitrogen concentration had no significant impact on the total lipid content of SCS-02. Increased light intensity can accelerate photosynthetic efficiency, leading to high carbon fixation efficiency. Storage lipids are the best option for the storage of excess energy [29]. Under high-light-intensity stress, the photoprotection of microalgae was activated to avoid excessive ROS generation (known to cause damage to the photosystem), which included reducing light absorption by decreasing light-harvesting antenna size [30]. The membrane lipids are the primary component of the thylakoid membranes in SCS-02. Thus, a reduction in thylakoid membranes led to a corresponding reduction in membrane lipids. An increase in storage lipids was accompanied by a decrease in membrane lipids; ultimately, the total lipid content remained relatively unchanged under different light conditions. Under low-light conditions, cells tend to synthesize more chloroplasts to enhance light absorption efficiency, resulting in an increased proportion of membrane lipids. These adaptations are consistent with the light acclimation mechanism observed in most plants [31]. High salinity increased the proportion of membrane lipids, likely due to salt-induced generation of superoxide, leading to membrane lipid peroxidation damage. In response, microalgae synthesized new membrane lipids to repair the thylakoid membrane [32]. High salinity inhibited the synthesis of storage lipids. DGAT (diacylglycerol acyltransferase), a key enzyme in TAG synthesis, might undergo down-regulation of its gene expression under high salinity, thereby suppressing storage lipid accumulation [18].

The proportions of ARA and EPA in SCS-02 ranged from 25.8% to 32.8% TFA and 3.7% to 6.9% TFA, respectively. This indicated that the ARA content was substantially higher than that of EPA, which contrasted with some previous reports where EPA reached approximately 20% TFA [8]. The discrepancy might be attributed to the different strains and cultivation periods. In this study, fatty acid composition was analyzed at the end of the cultivation period. EPA levels of the *P. purpureum* SCS-02 during the logarithmic-growth phase were significantly higher than those at the terminal phase, due to cellular aging in late culture [22]. This study demonstrated that the conditions conducive to ARA synthesis included low nitrogen, low phosphorus, high light intensity and high salinity, whereas the conditions conducive to EPA accumulation included high nitrogen, high phosphorus, low light intensity and high salinity. The conditions conducive to EPA synthesis were thus highly consistent with those conducive to membrane lipid synthesis. In *P. purpureum*, EPA was mainly distributed in MGDG (C16:0/C20:5), a major component of membrane lipids. Therefore, variations in membrane lipid content are closely related to changes in EPA levels [4]. The total EPA content decreased by 28% at highly saturating light for *Nannochloropsis oceanica* [27]. In contrast, ARA was mainly distributed in PCs (C16:0/C20:4) and TAGs (C16:0/C20:4/C20:4). Except for high salinity, the change in ARA content under other conditions (low nitrogen, low phosphorus and high light) aligned closely with changes in storage lipids, further supporting the finding that ARA existed within TAGs. The potential reasons why high salinity might increase ARA content were as follows: 1) high salinity disrupted cellular osmotic balance, and in response, SCS-02 increased the percentage of PUFAs in membranes to maintain membrane fluidity, as reported in *Dunaliella salina* [33]; 2) salt stress induced ROS accumulation, leading SCS-02 to enhance synthesis of PUFAs, which possessed strong reducing capacity and could neutralize ROS. A similar protective mechanism was observed in *Chlamydomonas reinhardtii*, which synthesizes PUFAs such as C18:3 (ALA) under salt stress [34].

Microalgae within the Eustigmatophyceae class, such as *Eustigmatos magnus*, *E. polyphem*, *E. vischeri*, *Vischeria helvetica*, *V. punctata* and *V. stellata*, are currently regarded as the most promising producers of EPA, with their EPA content generally exceeding 20% of TFA [35]. For instance, *E*. *vischeri* JHsu-01 and *V*. *punctate* IPPAS H-242 could achieve EPA contents of 20.6% and 20.2% of TFA, respectively [36,37]. In contrast, the maximum EPA content in SCS-02 used in this study was only 6.85% of TFA. SCS-02 can produce EPA, but its low yield makes it a poor candidate for EPA production. Notably, SCS-02 demonstrates a distinct advantage in ARA production. The highest ARA content in strain SCS-02 reached 33.12% of TFA in the present study. In comparison, the ARA contents in the aforementioned high-EPA-potential strains *V. punctate* IPPAS H-242 and *E. vischeri* JHsu-01 were only 3.90% and 7.85% of TFA, respectively [36,37]. An extensive analysis of fatty acid profiles across 2000 microalgal strains by Lang et al. (2011) revealed that, among all valid datasets, relatively high percentages of ARA were primarily found in genus *Porphyridium*, ranging from 10.3% to 44.5% of TFA [38]. *Porphyridium purpureum* SAG 1380-1e showed the highest content (44.5% of TFA), while *Porphyridium aerugineum* SAG 110.79 showed the lowest (10.3% of TFA), indicating substantial intraspecies variability in ARA accumulation within this genus. The ARA content of SCS-02 falls within the medium range among reported *Porphyridium* strains, suggesting its potential as a candidate for scalable ARA production.

The results of this study indicated that ARA and EPA were present in both membrane and storage lipids, with ARA being more abundant in storage lipids and EPA in membrane lipids. This finding was consistent with the lipidomics-based results reported by Li et al. (2024) [4]. The underlying reason lay in the distribution of Δ17DES and the low activity of Δ15DES in *P. purpureum*, which played a key role in the predominant distribution of EPA in MGDG [4]. Furthermore, culture conditions could change the distribution of ARA and EPA. High nitrogen, high phosphorus, low light and high salinity increased the proportions of ARA and EPA in membrane lipids, while low nitrogen, low phosphorus, high light and low salinity increased their proportions in storage lipids. This result was consistent with the results of the content of membrane and storage lipids. This indicated that cultivation strategies aimed at increasing membrane lipid content, such as nutrient-sufficient conditions, could increase the content of both ARA and EPA. In contrast, nutrient-limiting strategies, although proven efficient in increasing lipid content in oleaginous microalgae [16], had a negative impact on the enhancement of EPA and ARA content for *P. purpureum*. Therefore, to achieve high ARA and EPA production in *P. purpureum* SCS-02, it was best to provide sufficient nutrients and optimal culture conditions (light intensity and salinity) that enhanced membrane lipid synthesis. In contrast, stress conditions typically promoted the accumulation of polysaccharides while suppressing membrane lipid synthesis.

## 4. Materials and Methods

### 4.1. Algae Species

*P. purpureum* SCS-02 was used as the experimental material. This microalga strain was isolated from the South China Sea (16°56′ N, 112°16′ E) [8]. The sampling site is located in a tropical oceanic region, with a typical salinity of approximately 34 ppt and a sea surface temperature around 28 °C. *P. purpureum* SCS-02 is currently preserved at the South China Sea Institute of Oceanology, Chinese Academy of Sciences (Figure S1).

### 4.2. Experimental Design

Four culture conditions, including nitrogen and phosphorus concentrations, light intensity and salinity, were used to investigate the distribution of EPA and ARA in *P. purpureum* SCS-02. *P. purpureum* SCS-02 was cultured in a Ø3.0 cm × 60 cm glass column photobioreactor with ASW culture medium [8]. Illumination was provided by T8 fluorescent lamps (Philips; Suzhou, China) at 100–300 μmol photons m^−2^ s^−1^. The culture temperature was maintained at 25 ± 1 °C. CO_2_-enriched compressed air (1% CO_2_ in volume) was continuously bubbled into the photobioreactor to provide a carbon source. Each condition included three biological replicates. On day 16, samples were collected to measure biomass concentration, total lipid content, lipid composition and fatty acid content.

#### 4.2.1. Nitrogen Concentration

The experiment was conducted using KNO_3_ as the nitrogen source. The five KNO_3_ concentrations included 0.3, 0.5, 0.75, 1.5 (ASW medium) and 3.0 g L^−1^. Other medium components were consistent with the standard ASW medium [8]. Light intensity was 250 μmol photons m^−2^ s^−1^.

#### 4.2.2. Phosphorus Concentration

The experiment was conducted using K_2_HPO_4_ as the phosphorus source. The five K_2_HPO_4_ concentrations included 24, 40, 60, 120 (ASW medium) and 240 mg L^−1^. Other medium components were consistent with the standard ASW medium [8]. Light intensity was 250 μmol photons m^−2^ s^−1^.

#### 4.2.3. Light Intensity

The five light intensities included 100, 150, 200, 250 and 300 μmol photons m^−2^ s^−1^. The medium was the standard ASW medium [8].

#### 4.2.4. Salinity

The salinity of the culture medium was adjusted using NaCl. The five salinities included 5, 10, 20, 35 (ASW medium) and 50 ppt, with corresponding NaCl concentrations of 0, 7.7, 15.4, 27.0 and 38.5 g L^−1^, respectively. Other medium components were consistent with the standard ASW medium. Light intensity was 250 μmol photons m^−2^ s^−1^.

### 4.3. Determination Methods

#### 4.3.1. Determination of Biomass Concentration

A volume of 10 mL of the culture was filtered through a pre-dried filter membrane (0.45 μm) at 80 °C. The filter membrane with microalga cells was further dried at 80 °C to a constant weight. Biomass concentration was calculated according to the method of Li et al. (2019) [8].

#### 4.3.2. Determination of Total Lipid Content and Lipid Classification

The freeze-dried biomass (100 mg) was added to 3 mL of dimethyl sulfoxide/methanol solution (1:9, *v*:*v*). The mixture was extracted at 50 °C for 30 min, then further extracted in an ice bath for 60 min. After centrifugation, the supernatant was collected in pre-dried glass vials. The residue was then extracted with 6 mL of ether/hexane solution (1:1, *v*:*v*) in an ice bath for 120 min, followed by centrifugation to collect the supernatant in the same vial. Subsequently, 3 mL of pure water was added to the extracts, shaken and allowed to stand until separation. The upper organic phase was collected. The organic phase was dried under a gentle stream of nitrogen until constant weight, representing the total lipid content [4].

The total lipids were dissolved in chloroform–methanol solution (1:1, *v*:*v*) and separated into membrane lipids and neutral lipids using 500 mg of a Cleanert silica gel column (Agela Technologies; Tianjin, China). Storage lipids, mainly TAGs, were eluted with 10 mL of chloroform, followed by elution of membrane lipids (glycolipids and phospholipids) with 10 mL of methanol. To ensure the purity of individual lipid fractions, methanol-free chloroform must be selected for the elution step. In addition, the migration of bands should be closely monitored throughout the elution process. Neutral lipids exhibit a transparent orange color, whereas membrane lipids appear dark-green in color. This visual distinction allows for precise fraction collection and thus prevents cross-contamination of samples. Each fraction was collected in a small glass vial, dried under a stream of nitrogen to reduce volume, and transferred to a pre-weighed 2.5 mL plastic centrifuge tube. Further drying under nitrogen was performed until a constant weight was achieved for the weight of the individual lipid fractions [4].

#### 4.3.3. Determination of Fatty Acid Content

A volume of 2 mL of 2% H_2_SO_4_ in methanol and toluene (90:10, *v*:*v*) was added to the accurately weighed algal powder, storage lipids or membrane lipids. After flushing with argon gas, the mixture was incubated in a water bath at 80 °C with stirring for 1.5 h. Following incubation, 1 mL of pure water and 1 mL of n-hexane were added. The mixture was shaken to separate the phases, and the upper organic phase was transferred to a 1.5 mL sample vial. The solvent was dried under nitrogen gas, and then 1 mL n-hexane with C17:0 methyl esters was added. The contents of FAMEs were determined using GC-2014 gas chromatograph spectrometer with a flame ionization detector (Shimadzu; Kyoto, Japan), equipped with a 30 m fused silica DB-WAX capillary column (Agilent Technologies; Santa Clara, CA, USA). The temperature of the injection port was maintained at 260 °C. The column temperature was programmed from 140 °C to 240 °C at 10 °C min^−1^ with a hold of 5 min at 240 °C. High-purity argon was used as the carrier gas at a flow rate of 1.2 mL min^−1^. Individual peaks of FAMEs were identified through comparisons of retention times with 37 fatty acid standards (Nu-Chek-Prep; Elysian, MN, USA) [4].

The quantification of target fatty acid methyl esters (FAMEs) was performed as follows:

C_i_ = f_i_ × (C_s_ × A_i_/A_s_) (1)

where A_i_ and A_s_ are the peak areas of the target fatty acid methyl ester and the internal standard (C17:0 methyl ester), respectively; C_s_ is the known concentration of the internal standard; C_i_ is the concentration of the individual fatty acid in the final sample solution (1.0 mL); and f_i_ is the relative correction factor.

Individual fatty acid content (%DW) = C_i_ × v/m × 100% (2)

where m is the weight of the algal powder sample (mg), which was measured for fatty acid determination, and v is the final sample solution (1.0 mL).

#### 4.3.4. Yield Calculation

EPA yield (mg L^−1^) = m_16_ × E_16_ − m_0_ × E_0_ (3)

ARA yield (mg L^−1^): m_16_ × A_16_ − m_0_ × A_0_ (4)

where m_16_ is the biomass on day 16 (g L^−1^); m_0_ is the biomass on day 0 (g L^−1^); E_16_ is the EPA content on day 16 (% DW); E_0_ is the EPA content on day 0 (% DW); A_16_ is the ARA content on day 16 (% DW); and A_0_ is the ARA content on day 0 (% DW).

### 4.4. Statistical Analysis

All means and standard deviations presented in the figures were calculated based on three biological and three measurement replicates. Data analysis was conducted using SPSS 18.0 software, employing one-way analysis of variance (ANOVA). Differences between sample means were evaluated using the least significant difference (LSD) test at a 0.05 confidence level.

## 5. Conclusions

The optimal culture conditions for biomass accumulation in SCS-02 were a nitrogen concentration of 0.75 g L^−1^, phosphorus concentration of 240 mg L^−1^, light intensity of 250–300 μmol photons m^−2^ s^−1^ and salinity of 50 ppt. High nitrogen concentration, high phosphorus concentration and high salinity could promote the synthesis of membrane lipids but were unfavorable for storage lipid synthesis, whereas high light intensity favored storage lipid synthesis over membrane lipids. ARA was predominantly distributed in storage lipids, whereas EPA was mainly distributed in membrane lipids. Culture conditions could change the distribution of ARA and EPA between storage and membrane lipids. High nitrogen, high phosphorus, low light intensity and high salinity increased the proportion of ARA and EPA in membrane lipids, while low nitrogen, low phosphorus, high light intensity and low salinity enhanced their proportion in storage lipids. In conclusion, to obtain oil rich in both ARA and EPA, it is advisable to provide relatively abundant nutrients for *P. purpureum*.

## Figures and Tables

**Table 1:** The content and yield of EPA and ARA in *Porphyridium purpureum* SCS-02.

Treatments		ARA		EPA	
		Content (%DW)	Yield (mg L^−1^)	Content (%DW)	Yield (mg L^−1^)
Nitrogen concentration (g L^−1^)	0.30	3.53 ± 0.21 ^a1^	13.28 ± 0.16 ^e2^	0.47 ± 0.01 ^c3^	1.77 ± 0.01 ^d4^
	0.50	3.37 ± 0.17 ^a1^	15.19 ± 0.49 ^d2^	0.48 ± 0.01 ^c3^	2.16 ± 0.02 ^c4^
	0.75	3.43 ± 0.10 ^a1^	19.37 ± 0.21 ^a2^	0.51 ± 0.02 ^b3^	2.86 ± 0.03 ^b4^
	1.50	3.40 ± 0.14 ^a1^	17.67 ± 0.30 ^b2^	0.55 ± 0.02 ^b3^	2.86 ± 0.04 ^b4^
	3.00	3.43 ± 0.27 ^a1^	18.35 ± 0.17 ^c2^	0.61 ± 0.01 ^a3^	3.28 ± 0.05 ^a4^
Phosphorus concentration (mg L^−1^)	24	4.43 ± 0.30 ^a1^	14.12 ± 0.30 ^c2^	0.57 ± 0.02 ^b3^	1.81 ± 0.03 ^e4^
	40	4.03 ± 0.12 ^b1^	17.91 ± 0.43 ^b2^	0.59 ± 0.01 ^b3^	2.60 ± 0.03 ^d4^
	60	3.83 ± 0.10 ^c1^	18.55 ± 0.20 ^a2^	0.57 ± 0.02 ^b3^	2.77 ± 0.05 ^c4^
	120	3.64 ± 0.09 ^d1^	18.84 ± 0.41 ^a2^	0.59 ± 0.01 ^b3^	3.05 ± 0.04 ^b4^
	240	3.26 ± 0.05 ^e1^	17.86 ± 0.77 ^b2^	0.64 ± 0.00 ^a3^	3.49 ± 0.16 ^a4^
Light intensity (μmol photons m^−2^ s^−1^)	100	3.60 ± 0.08 ^a1^	14.00 ± 0.31 ^c2^	0.82 ± 0.01 ^a3^	3.19 ± 0.16 ^b4^
	150	3.58 ± 0.18 ^a1^	17.56 ± 0.60 ^b2^	0.69 ± 0.02 ^b3^	3.38 ± 0.14 ^a4^
	200	3.54 ± 0.11 ^a1^	18.03 ± 0.38 ^b2^	0.59 ± 0.02 ^c3^	2.99 ± 0.11 ^c4^
	250	3.57 ± 0.08 ^a1^	18.38 ± 0.40 ^b2^	0.56 ± 0.02 ^c3^	2.87 ± 0.07 ^c4^
	300	3.71 ± 0.24 ^a1^	19.47 ± 0.60 ^a2^	0.54 ± 0.02 ^c3^	2.84 ± 0.03 ^d4^
Salinity (ppt)	5	4.21 ± 0.17 ^a1^	7.62 ± 0.19 ^d2^	0.61 ± 0.02 ^a3^	1.09 ± 0.03 ^d4^
	10	3.98 ± 0.13 ^b1^	11.44 ± 0.07 ^c2^	0.61 ± 0.01 ^a3^	1.76 ± 0.01 ^c4^
	20	3.63 ± 0.11 ^c1^	15.90 ± 0.41 ^a2^	0.61 ± 0.01 ^a3^	2.66 ± 0.04 ^a4^
	35	3.03 ± 0.12 ^d1^	15.57 ± 0.37 ^a2^	0.49 ± 0.02 ^b3^	2.51 ± 0.03 ^b4^
	50	2.50 ± 0.02 ^e1^	13.32 ± 0.49 ^b2^	0.49 ± 0.01 ^b3^	2.60 ± 0.12 ^b4^

DW: Dry weight; EPA: eicosapentaenoic acid; ARA: arachidonic acid. The values shown are the averages of three biological replicates and three technical replicates ± standard deviation. Different letters denote significant differences among the values of content and yield on the different treatment groups (a1–e1: ARA content; a2–e2: ARA yield; a3–c3: EPA content; a4–e4: ARA yield; level of significance, *p* < 0.05).

## Data Availability

The data presented in this study are available on request from the corresponding author due to legal reasons.

## Supplementary Materials

The following supporting information can be downloaded at https://www.mdpi.com/article/10.3390/md24030114/s1: Figure S1. Microscopic images of *Porphyridium purpureum* SCS-02.

## Abbreviations

The following abbreviations are used in this manuscript:

ARA	Arachidonic acid
EPA	Eicosapentaenoic acid
TAG	Triacylglycerols
MGDG	Monogalactosyldiaclyglycerol
DGDG	Digalactosyldiaclyglycerol
SQDG	Sulfoquinovosyldiacylglycerol
PC	Phosphatidylcholine
PG	Phosphatidylglycerol
DW	Dry weight
TFA	Total fatty acids
TL	Total lipids
