# Supplementary material for: Effects of Different Culture Conditions on the Synthesis and Distribution of Polyunsaturated Fatty Acids (EPA and ARA) in Porphyridium purpureum
Source: Mar Drugs. 2026 Mar 19;24(3):114. doi: 10.3390/md24030114 (PMC13028394; doi:10.3390/md24030114)
Supplement: Supplementary file 1 [file marinedrugs-24-00114-s001.zip › marinedrugs-4147538-supplementary.pdf]

Supplementary Data

Porphyridium purpureum SCS-02 is a unicellular red microalga belonging to the phylum Rhodophyta, class Bangiophyceae, order Bangiales, family Porphyridiaceae, and genus *Porphyridium*. The algal cells are spherical, with a diameter of approximately 8–15 μm, and lack a rigid cell wall. Each cell contains a large, star-shaped chromatophore rich in phycoerythrin, which gives the alga its distinctive red color; this stellate chromatophore is a characteristic feature of the genus.

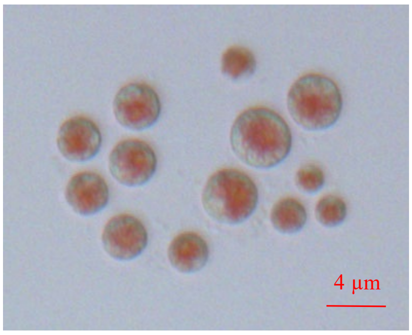

Figure S1. Microscopic images of *Porphyridium purpureum* SCS-02.
